# The effect of eraser sampling for proteomic analysis on Palaeolithic bone surface microtopography

**DOI:** 10.1038/s41598-021-02823-w

**Published:** 2021-12-08

**Authors:** Virginie Sinet-Mathiot, Naomi L. Martisius, Ellen Schulz-Kornas, Adam van Casteren, Tsenka R. Tsanova, Nikolay Sirakov, Rosen Spasov, Frido Welker, Geoff M. Smith, Jean-Jacques Hublin

**Affiliations:** 1 Department of Human Evolution, Max Planck Institute for Evolutionary Anthropology, Leipzig, Germany; 2 Department of Anthropology, The University of Tulsa, Tulsa, OK USA; 3 Department of Cariology, Endodontology and Periodontology, University of Leipzig, Leipzig, Germany; 4 National Institute of Archaeology with Museum, Bulgarian Academy of Sciences, Sofia, Bulgaria; 5 Archaeology Department, New Bulgarian University, Sofia, Bulgaria; 6 Section for Evolutionary Genomics, Globe Institute, University of Copenhagen, Copenhagen, Denmark; 7 Collège de France, Paris, France

**Keywords:** Archaeology, Proteomics

## Abstract

Bone surface modifications are crucial for understanding human subsistence and dietary behaviour, and can inform about the techniques employed in the production and use of bone tools. Permission to destructively sample such unique artefacts is not always granted. The recent development of non-destructive proteomic extraction techniques has provided some alternatives for the analysis of rare and culturally significant artefacts, including bone tools and personal ornaments. The Eraser Extraction Method (EEM), first developed for ZooMS analysis of parchment, has recently been applied to bone and ivory specimens. To test the potential impact of the EEM on ancient bone surfaces, we analyse six anthropogenically modified Palaeolithic bone specimens from Bacho Kiro Cave (Bulgaria) through a controlled sampling experiment using qualitative and 3D quantitative microscopy. Although the overall bone topography is generally preserved, our findings demonstrate a slight flattening of the microtopography alongside the formation of micro-striations associated with the use of the eraser for all bone specimens. Such modifications are similar to ancient use-wear traces. We therefore consider the EEM a destructive sampling approach for Palaeolithic bone surfaces. Together with low ZooMS success rates in some of the reported studies, the EEM might not be a suitable approach to taxonomically identify Pleistocene bone specimens.

## Introduction

Bone is one of the most common archaeological remains recovered from Palaeolithic sites. Analysis of these remains can provide insights into human subsistence behaviour, dietary practices and site formation processes. Bone surface modifications are informative in this regard, particularly to retrace the taphonomic history of a bone fragment or to understand the manufacturing process and the potential use of a worked bone^1,2^. The taxonomic identification of these specimens through morphological assessments then becomes crucial^3^, in particular related to raw material selection at the species or skeletal element level. Worked bones such as bone tools represent technological innovation during human evolution^4–8^, and the selection of raw material reflects behavioural choices and, potentially, also the function of the tool^9^. Tool production can be driven by opportunistic bone selection made among the available faunal assemblage on-site resulting from food consumption^10,11^. In such a case, species composition and skeletal representation of a bone artefact assemblage could reflect species (and element) composition at the archaeological site or the faunal community present on the landscape. Alternatively, the raw material choice of a specific taxa and/or bone element can be based on the biomechanical properties required by the function of the tool, and knowledge thereof^12–14^, or can be driven by behavioural choices related to cultural and/or symbolic meanings associated with a specific taxa (and/or element)^15,16^. Therefore, species determination is greatly informative for our understanding of their production and the culturally-mediated behaviours associated with technological choices.

The vast majority of bone material, including bone tools, found on Palaeolithic sites are highly fragmented due to various taphonomic processes and prevent the taxonomic assessment of these bone specimens based on morphology. Moreover, taxonomic assignments of bone artefacts based on visual inspection of the external appearance are rendered difficult by the removal of morphological features during the fabrication of the tools or, subsequently, during tool use^13,16,17,18^. Often, bone artefacts are analyzed without knowing species identity, or lack specific taxonomic assignment; e.g. are assigned to broad taxonomic groups such as large- or medium size classes based on bone thickness, or even in relation to the most frequent species within the morphologically identifiable portion of the assemblage. Such assignments are not necessarily correct^19^, and lack taxonomic precision.

To overcome these obstacles, researchers are using biomolecular approaches like palaeoproteomics, in particular Zooarchaeology by Mass Spectrometry (ZooMS)^20,21^, ancient DNA analysis, and high-resolution CT scanning of bone histological thin sections to assess raw material selection and behavioural aspects associated with  the artefact^22,23^. In particular proteomic peptide mass fingerprinting such as using ZooMS, has been applied frequently to the study of archaeological bone artefacts^18,24–29^ and provides a precise taxonomic identification based on the analysis of the bone protein collagen type I^20^. Collagen type I survives beyond the temporal range of ancient DNA^30^ and provides specimen-specific information about molecular diagenesis^31,32^. However, despite the small sample size required in its traditional version, such destructive sampling is problematic for the analysis of rare, culturally significant and highly valuable archaeological artefacts.

With the expansion of ZooMS applications, recent studies have focused on developing non-destructive collagen extraction techniques, which began in 2015 using an eraser method initially applied to thirteenth century parchments^33^. This significant advance in biocodicology has unlocked the development of biomolecular analysis of parchments^34,35^, and has been replicated for the extraction of DNA from herbarium specimens^36^. The Polyvinyl Chloride (PVC) eraser method consists of rubbing a soft polymer eraser on the surface of an organic tissue^33^. It is generally believed that the friction caused by the eraser rubbing generates a triboelectric charge between the organic surface and the eraser, releasing protein from the bone surface that binds to the eraser waste^32^. Alternative non-destructive sampling protocols also employ static electricity to extract proteins from sample surfaces^13,37^. PVC erasers are widely used by conservators as a conventional conservation treatment for cleaning parchment and paper surfaces^38^. This method does not require specialized equipment and the protein extract can be obtained on-site without transporting the specimen. The Eraser Extraction Method (EEM) is one of the non-invasive strategies called “eZooMS” (electrostatic ZooMS) and has recently been extended to various archeological materials such as bone and ivory^25,37,39^.

Using EEM to assess taxonomic identification allows for the possibility of non-invasive analyses while preserving the integrity of these specimens^37^. However some specialists have also shown that undertaking surface cleaning treatment using an eraser on paper can be abrasive to the specimen surface, particularly through the removal of fibers^40–42^. Likewise, due to its potentially abrasive nature, we hypothesize that the use of PVC erasers on bone surfaces might modify macro- and microscopic features of the surface topography. We assume that it could remove large-scale macroscopic traces, such as the cross sectional shape of cut marks at mm-scale. In addition, it could potentially alter the surface roughness of the bone microtopography and produce small-scale microscopic use-wear traces such as micro-striations at µm-scale. This would result in unintentionally modified surfaces of the archaeological material, similar to natural processes that can produce pseudotools^43^, but also may cover or overprint ancient traces. Such aspects have recently received increased attention, as they can be informative on taphonomic processes and human behaviour^44–47^.

We note that although the advantages of the EEM for biomolecular analysis are clear, its impact on bone surfaces and bone surface modifications has not been assessed. To address this caveat, we characterize any potential modifications to archaeological bone surfaces resulting from EEM sampling. In addition, we assess the implications of such modifications relating to subsequent archaeological analyses like use-wear or bone surface modification analysis. To describe the modifications, first, we measure downward forces applied during EEM, and second, analyze bone surfaces with and without cut marks before and after EEM at different scales using qualitative (digital microscope, 2D) and quantitative microscopy (confocal disc-scanning microscope, 3D)^13,48^.

## Methods

### Bone selection and sampling location

**Figure 1:**
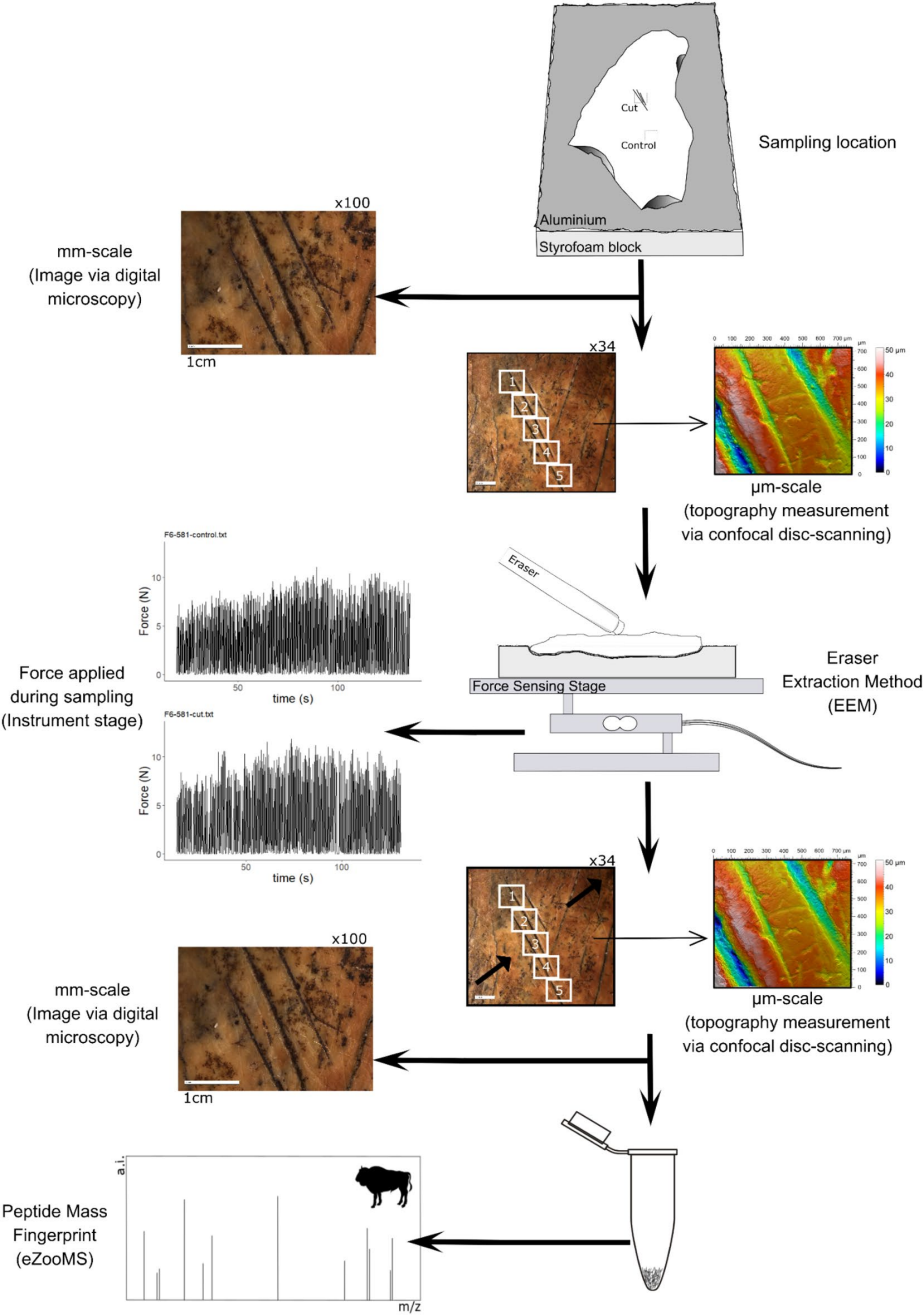
Description of the experimental workflow. The location of ROIs (cut and control) was defined on each bone specimen included in the study. The macro- and microscopic surface topography of the bone surface of each area was visually inspected using photos by digital microscopy (ZEISS, Smartzoom 5) and measurements by confocal disc-scanning microscopy (μsurf mobile, Nanofocus AG) before and after EEM. Cut and control areas were sampled using EEM while the downward force applied during sampling was measured via an instrumented stage. Each sample collected was analysed through peptide mass fingerprinting (n = 12). Animal silhouette is not to scale and derives from http://phylopic.org.

We selected six archeological bone specimens from Bacho Kiro Cave (Dryanovo, Bulgaria), which were directly dated to approx. 45,000 calBP^49^. These large long bone fragments have previously been taxonomically identified as *Bos/Bison* through ZooMS using destructive sampling. We determined by visual inspection that they show good surface preservation with clear anthropogenic traces including cut marks, marrow fractures or damage from reshaping lithic tools^49,50,51^. We defined two regions of interest (ROI) on each bone surface, represented by two squares of 1 × 1 cm each. One ROI was located on top of a butchery trace with cut marks (cut area), and another ROI was located on an unmodified surface (control area) (Fig. 1).

### Eraser sampling protocol

The bone specimens were placed into a styrofoam block that fit the shape of the bone fragment so that a standardised position of the bone specimen was maintained throughout the sampling process. The eraser sampling was done by one individual (VSM) to avoid any potential inter-individual variability and followed details provided by^33^. All samples were obtained through the rubbing of a sterile PVC eraser on a bone surface. An eraser holder and eraser sticks (Staedtler Mars plastic 52855) were used. The erasers were wiped with ethanol (Roth, 99.98%) and wrapped separately in aluminium foil before sampling. The holder was cleaned with ethanol between each sampling event. The eraser piece was replaced after each sampling event. Sampling was done with unidirectional movements^33^. Eraser movements were orientated perpendicular to the cut mark, limited to a duration of 2 min in order to standardize the experiment for all specimens. This duration is, in our experience, generally sufficient to generate the recommended amount of eraser scrubbings, equivalent to 20 μl^52^. The eraser wastes were collected in aluminium foil placed directly underneath the bone specimen and transferred into labeled LoBind tubes (Eppendorf).

### ZooMS analysis

The eraser waste obtained during sampling was analyzed through previously described ZooMS protocols^20,33,53^. In short, eraser waste was centrifuged for 1 min and incubated with 100 µL of 50 mM ammonium bicarbonate solution (NH_3_CO_3_) for 1 h at 65 °C. 50 µL of the supernatant was transferred into a new Lo-Bind tube (Eppendorf). 1 µL of trypsin (0.5 µg/µL, Promega) was added to the gelatin extract and incubated at 37 °C for 18 h. After digestion, each sample was acidified using 1 µL of 10% TFA and cleaned on C18 ZipTips (Thermo Scientific). Subsequently, the eluted peptides were spotted in triplicate on a MALDI Bruker plate with the addition of matrix solution (CHCA). MALDI-TOF MS analysis was conducted at the IZI Fraunhofer in Leipzig and spectra were identified in comparison to a database containing peptide marker masses for all medium to larger sized mammalian genera in existence in Europe during the Pleistocene^53^. To compare spectral quality between sample extracts, we calculated signal-to-noise ratios (S/N) for a selected set of peptides using MALDIquant, including baseline removal and peak picking^54^.

In order to assess any potential contamination by non-endogenous peptides, we performed a set of blank extractions, consisting of empty tubes, alongside the rest of the samples in order to exclude any potential protein contamination during laboratory extraction. The MALDI-TOF MS spectra obtained show no collageneous peptides, demonstrating that the taxonomic identification does not derive from laboratory contamination. Secondly, all spectra were checked against known contaminant peptide masses such as human keratin and any matching peaks were excluded from further analysis.

### Measuring the force applied using a force sensing stage

**Figure 2:**
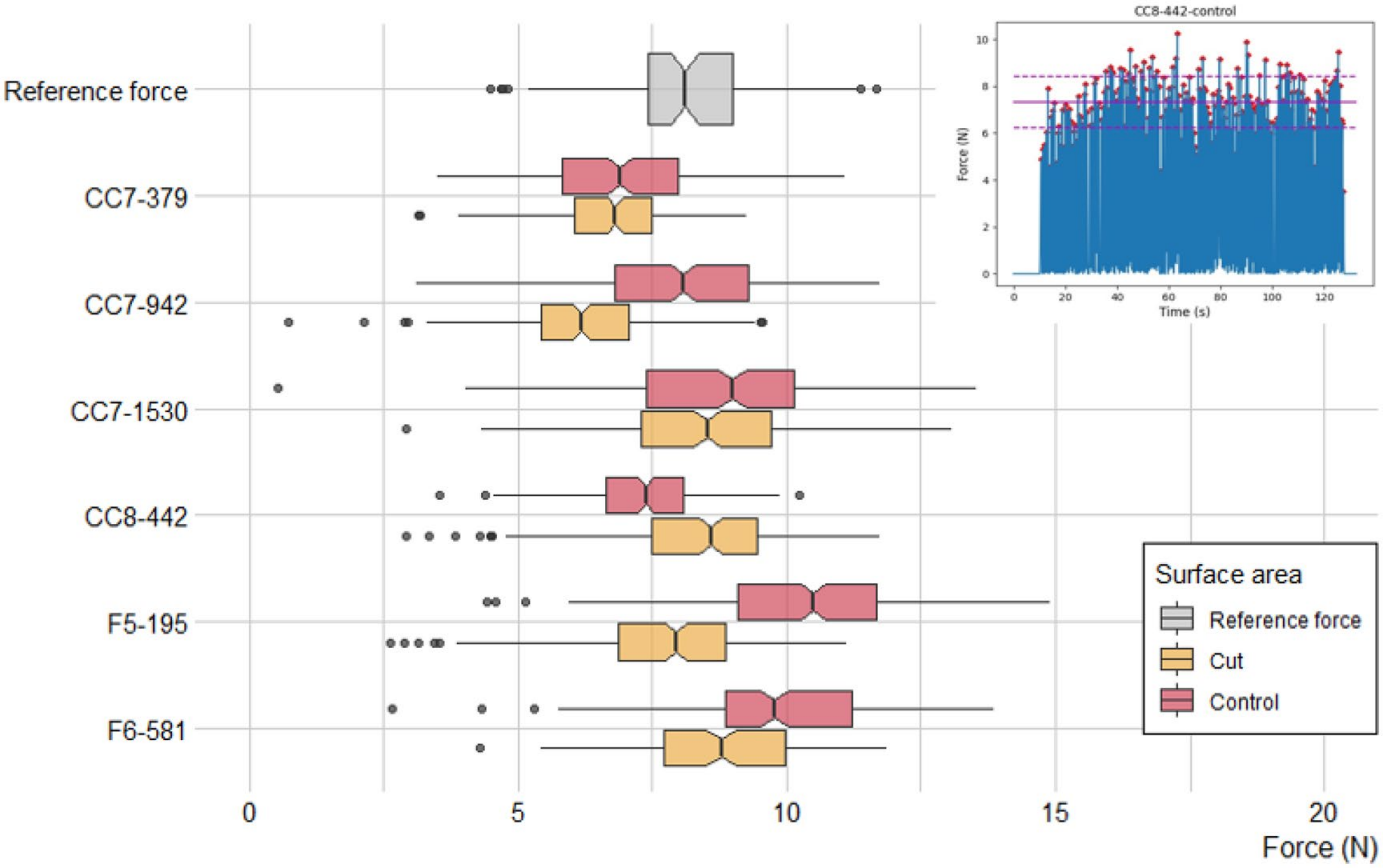
Distribution of the peak forces applied during the EEM of each sampling cut area (in yellow) and control area (in red) for each bone specimen. The reference peak force measurements (eraser on paper) is shown in grey. The insert in the top right is an example of how peak forces were acquired from force data. The mean peak force (red line) for each surface area consists of the maximum force values (red markers) for each eraser and bone contact during the 2 min of the EEM event. Dashed lines equal to + 1 and − 1 SD.

The intensity of force and the rate of erasing movements during the experiment were recorded by a self-made instrumented stage that reads the force applied to bones. The stage is composed of a load cell (5 kg CZL635, Tinkerforge, Stukenbrock, Germany) mounted between two 1 cm thick aluminium plates. The load cell has a precision of 0.05% across its full range of 49.04 N (equating to 5 kg). The load cell output is amplified by a HX711 load cell amplifier (SparkFun, Colorado, USA), which is then connected to an Arduino Nano microcontroller (Arduino, Ivrea, Italy). The stage interfaces with a laptop computer through two custom programs: one program to read the forces and one to calibrate the load cell. Force data is recorded at a rate of 10 Hz. The force sensing stage was secured to the laboratory bench using two screw clamps to limit the movement or vibrational noise. Before each sampling session the load cell calibration was checked using a series of known weights (Supplementary Fig. S1). In order to provide a reference point for comparison, the force applied during the erasing of pencil traces from paper was measured. Peak forces, above a threshold of 0.5 N, for each contact event between eraser and bone surface were identified using the SciPy library in Python 3.9 and the “find_peaks” function. This allowed the identification of the frequency of contact events and the peak force associated with each event (Fig. 2).

### Qualitative microscopy (digital microscopy)

Cut and control areas on each bone specimen were assessed using a digital microscope (Smartzoom 5 with lens PlanApoD 1.6/0.1FWD 36 mm, Zeiss) and photo images were taken using the Smartzoom 5 software version 1.4 (Zeiss), in a standardised manner, before and after EEM using a digital zoom of × 34/100 (Supplementary Fig. S2). The magnification was standardised for all bone specimens and ROIs, and the position and orientation of the bone specimens were normalized by their inclusion into their styrofoam block. The intensity, orientation and inclination of the light source were kept consistent across samples. The focus was adjusted manually and the segmented ringlight was used on different fixed positions for all images (full, top, bottom, right and left ringlight). In order to obtain the extended depth of field, the lower and higher focal plane were assigned manually. Each ROIs was assessed at two magnifications (× 34 and × 100) before and after eraser sampling. Visual inspection was conducted by one individual (VSM), and photo images were qualitatively described using the following terminology: the general appearance and rugosity of the surface were compared before and after EEM, the location and morphology of the cut marks were reported, as well as the presence of residues and their distribution on the surface. Surface reflectivity was addressed through a visual comparison of the intensity and localisation of the most reflective area. Removal/creation of traces appearing after the use of eraser was described including the indication of their orientation.

### Quantitative microscopy (confocal disc-scanning microscopy)

In order to assess the surface texture of the bone microtopography, each bone specimen was scanned with a confocal disc-scanning microscope (μsurf mobile, Nanofocus AG, Oberhausen, Germany), using a 20 × lens (numerical aperture = 0.4, field of view = 0.8 mm^2^). The confocal disc-scanning was done by one individual (ESK) to avoid any potential inter-individual variability and the measuring procedure followed details provided by^48,55^. For the two ROIs on each specimen, we ensured the same scanned position before and after EEM, through the creation of two reference points on a piece of tape placed on the surface of the bone (an incised X and a drawn point). Five scans within both ROIs were taken in sequence along the longitudinal axis of each cut mark (cut area). The area without cut marks (control area) was scanned in a similar manner along the same axis for each bone specimen. Scans were reviewed for quality and accepted for further study if 95% or more of the surface points were measured. Those with lesser accuracy were re-measured by altering exposure, brightness, gain, or pitch values until 95% of the surface was captured.

Within MountainsMap Premium v. 8.1.9369 Analysis software by Digital Surf (Besançon, France), meshed axiomatic 3D models of each scan were constructed using the following procedures: extract layers (topography layer), leveling (LS-plane), outlier removal (isolated outlier removal, with normal strength, and fill in non-measured points) following the pre-processing algorithms as described previously^48,55–57^. To compare the same scan area before and after eraser sampling, the following three pre-processing operators were included in the workflow: build series of surfaces (settings: copy after, use X/Y-offset, T-axis spacing 2), shift surface (settings: fixed reference studiable, offset settings manually defined, intersection set as kept area), and extract surfaces (settings: all surfaces of the series). One set of the paired before and after scans (second measurement location in the control area of specimen CC7-379) was removed at this point due to minimal overlap in surface portions. ISO 25178 parameters were then calculated from the S-L (roughness surface) using a filter set of an S-filter (Robust Gaussian polynomial of second order, 0.8 µm) and an L-filter (Robust Gaussian polynomial of second order, 0.008 mm).

We selected four ISO 25178 parameters^58^ for quantitative analysis based on previous experience assessing diagnostic alterations to bone microtopography: arithmetic peak height (*Sa*), arithmetic mean peak curvature (*Spc*), closed hill area (*Sha*), and upper material ratio (*Smrk1*)^48,57^ (Supplementary Fig. S3). *Sa* is a height parameter that represents the distribution of heights along the z-axis of the surface in comparison to the arithmetic mean value of the surface. Thus, higher values indicate a surface with greater variation or roughness along the z-axis. *Spc* is a feature parameter that represents the arithmetic mean curvature of the peaks of the surface. Higher values indicate more pointed peak forms. Another feature parameter, *Sha,* represents the average hill area that is not connected to the boundary edge at a given height of the material ratio. Higher values indicate a surface with large cross sections in the upper portion of the surface. *Smrk1* is a functional parameter calculated from the material ratio curve of the distribution of surface depths and represents the uppermost portion of the surface. Higher values indicate a plateaued surface.

### Statistical analysis of the ISO 25178 surface texture parameters

We employed Bayesian modeling following previous protocols^48,57^. To stabilize the variances and distributions, the ISO 25178 parameters were log transformed (Table 1, Supplementary Table S1). The statistical model for the observations Y, a matrix of p = 4 columns (log-transformed ISO 25178 parameters) and n = 59 rows (3D models), is a multivariate mixed model of the form $Y=XB+ZU+E$; where XB represents the fixed effects, ZU the random effects, and E the residual error. ZU captures idiosyncratic bone *specimens* and *measurement location* effects. U is a 65 × 4 matrix of random intercepts; each column of U contains 6 unique *specimen* effects and 59 unique *measurement location* effects. The four ISO 25178 parameters are represented by a column of U. Z is a 118 × 65 matrix of zeros and ones, which indicates the *specimen* and *measurement location* of each scan. The dimensions of X and B depend on the number of fixed effects. We fit two models of increasing complexity with different effects. M0 includes one fixed effect *area*, while M1 includes the additional effect *erasing*. We compared leave-one-out cross-validation (LOO) scores^59^, which found design (M1) to generate model predictions best (Supplementary Table S2). This indicates that there is statistical support for change in at least one ISO 25178 parameter related to eraser use on the bone surfaces. For M1, B is a 3 × 4 matrix of fixed effect *area* (control, cut) and *erasing* (after) and an intercept for each surface texture parameter. Design matrix X is a 118 × 3 matrix of zeros and ones. E is a 118 × 4 residual matrix. Therefore, M1 is a multilevel, multivariate Bayesian model with fixed effects (*area* and *erasing*), random effects (*specimen* and *measurement location*), and *error* (Supplementary Table S2).

**Table 1:** Descriptive statistics of the raw ISO 25178 data and model ratios based on posterior effects of each fixed effect in the model after and before EEM for each surface texture parameter including 95% credibility intervals. Intervals that include 1.00 indicate that the parameter ratio values are not well distinguishable prior to and after EEM.

ISO 25178 parameter	Descriptive statistics (raw values)		Model ratios (based on log-transformed values)		
	BEFORE Mean (SD) [Unit]	AFTER Mean (SD) [Unit]	2.5% Quantile	Median	97.5% Quantile
*Sa*	0.60 (0.32) [µm]	0.59 (0.32) [µm]	0.97	0.99	1.01
*Spc*	2.71 (1.29) [1/µm]	2.69 (1.37) [1/µm]	0.96	0.98	1.01
*Sha*	123.50 (59.73) [µm^2^]	127.85 (67.71) [µm^2^]	0.95	1.00	1.04
*Smrk1*	19.87 (0.95) [%]	20.24 (0.82) [%]	1.01	1.02	1.03

We applied a goodness of fit check to ensure that *specimen* and *measurement location* random effects were adequately modeled using multivariate Gaussian distributions. For additional model details see Martisius et al.^48^. We estimated effects by a Hamiltonian Markov-chain Monte Carlo method, using the library rstan version 2.21.2^60^ of the statistical computing language R version 4.1.0^61^. We allowed a 2000-iteration warm-up for four chains generating 1000 parameter samples per chain resulting in 4000 posterior samples for inference. We examined scaled and squared Mahalanobis distances between observations and predicted values to check for goodness of fit, and compared these distances to theoretical quantiles of the F-distribution^62^ using a quantile–quantile plot (Supplementary Fig. S4).

## Results

### ZooMS analysis

eZooMS analysis shows preserved collagen type I in each sample and, at a minimum, provides a MALDI-TOF MS spectrum containing two peptide markers (COL1α1 508–519 and COL1α2 978–990). In our spectra, we observe a systematic absence of peptides of higher molecular weight, in particular markers COL1α2 454–483, COL1α1 586–618 and COL1α2 757–789, which are absent in all eZooMS spectra, and COL1α2 793–816 present in one spectra (Supplementary Table S3 + Supplementary Fig. S5). This is in contrast to the previously obtained spectra from the same specimens, which were generated using a destructive sampling approach, and where such heavier peptide markers are present. The general absence of heavy m/z peptides within non-destructively extracted samples has also been observed in previous eZooMS studies^13,37^. The assessment of the peak intensity between comparable samples illustrates a signal considerably lower for the eraser samples than for the bone fragments of the same specimens analysed through ZooMS. The signal-to-noise ratio (S/N) of the three dominant peptide markers (COL1α1 508–519, COL1ɑ2 978–990, and COL1ɑ2 484–498) of the eZooMS samples show, in the case of COL1α1 508–519, COL1ɑ2 978–990, lower to similar values compared to the bone samples with the exception of specimen CC7-1530. The S/N values for the peptide marker COL1ɑ2 484–498 are consistently higher for the bone samples compared to the values obtained from eZooMS (Supplementary Fig. S6).

Each spectrum obtained with the EEM produced a taxonomic identification in agreement with those previously made using a destructive sample of the same bone specimens^50^. However, eZooMS identifications are broader due to the absence of the higher mass peptides, resulting in identifications as *Bos/Bison/Ovibos* instead of *Bos/Bison*. The mass 1208 m/z corresponds to the peptide COL1ɑ2 978–990 present in *Bos* sp*., Bison* sp*., *and* Ovibos* sp. This peptide was systematically identified within each MALDI-TOF MS spectra and, despite the absence of higher mass peptides, permitted such a specific taxonomic attribution. The genera *Bos* sp. and *Bison* sp. cannot be separated from each other based on standard ZooMS peptide markers, while in our case the peptide COL1ɑ1 586–618 is absent and also prevents the eZooMS identifications to exclude *Ovibos* sp. as a possibility.

### Force sensing

We analysed the peak forces applied during each EEM event (n = 12) and compared these to a reference set of peak forces obtained when erasing a pencil mark from paper (Fig. 2). All forces remain low at less than 15 N of force, the mean peak force applied to the bones during the EEM (8.12 N, ± 1.21 N) is comparable to the mean peak forces applied during the test on paper (mean = 8.15 N, ± 1.44 N). The peak forces applied to the bones were significantly different between specimens and sampled areas (Kruskal Wallis test, p-value < 0.05), but without a clear pattern or direction. However, these significant differences amount to only very small deviations in the mean peak force between conditions, all occurring within less than 5 N of each other. With this in mind we assert that the peak forces recorded throughout this experiment are representative of real-world erasing events and are broadly comparable in each experimental condition.

Sample weights consisting of the eraser wastes collected during the experiment ranged from 0.5 to 15.6 mg and were consistently higher for the control area compared to the cut area (Supplementary Table S3). However we observe no significant difference of the mean peak force applied between cut and control areas (t(10) = 0.41, p = 0.7) and also no significant difference between the mean number of eraser movements applied to cut area compared to control area (t(10) = 0.58, p = 0.6). There is no correlation between generated sample weight and generated force during the EEM procedure either, nor between generated force and the signal-to-noise ratio (S/N) of three low-weight peptide markers (COL1α1 508–519, COL1ɑ2 978–990, and COL1ɑ2 484–498) across samples (Supplementary Fig. S7). We therefore conclude that the force applied in these experiments has had no measurable influence on our eZooMS spectral quality.

### Qualitative microscopy

**Figure 3:**
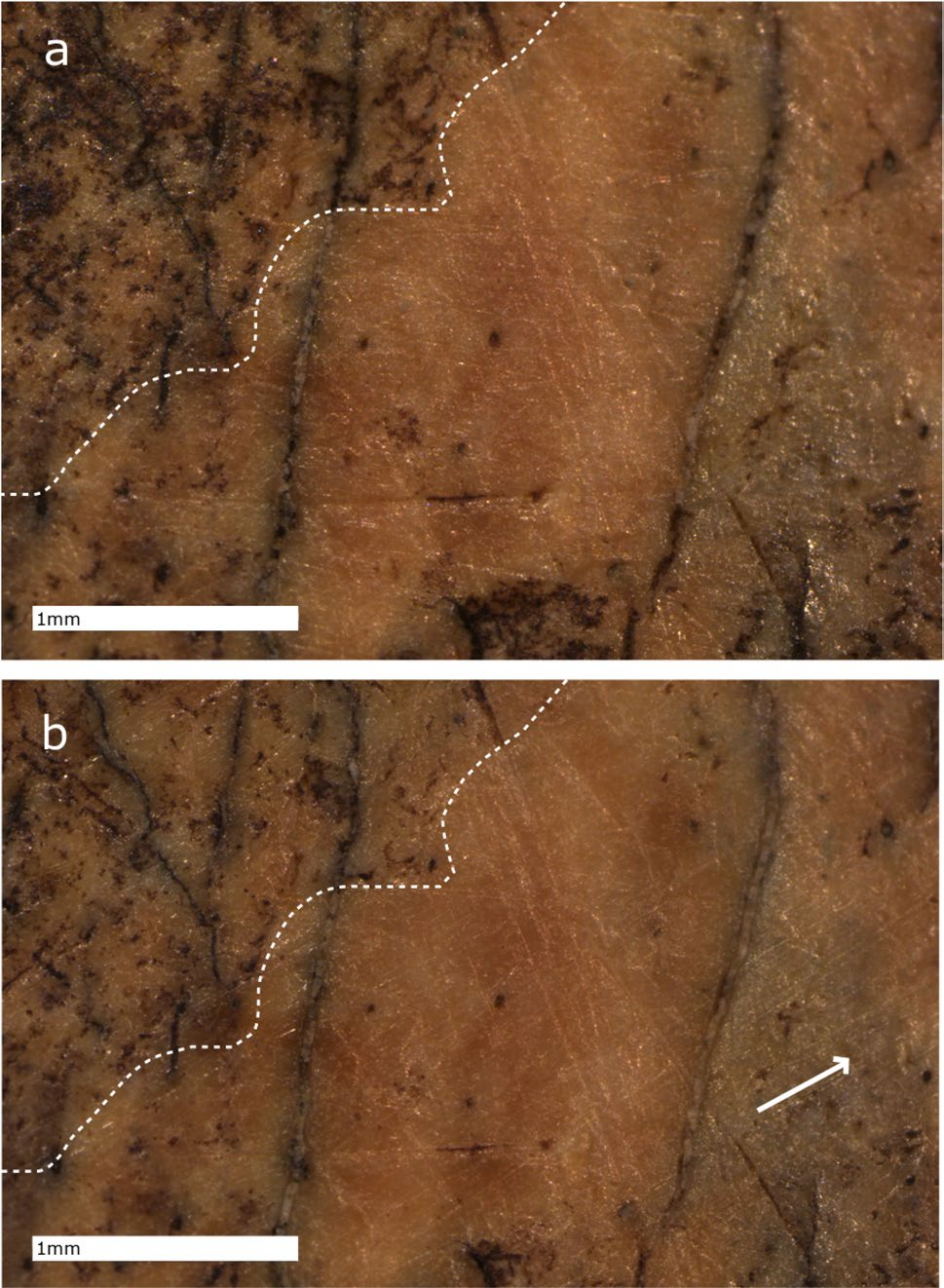
Micrographs of the control area of CC7-379 using automated digital microscopy (ZEISS, Smartzoom 5), (**a**) before the use of EEM, (**b**) after the use of EEM. The white arrow highlights the orientation of the micro-striations which follow the orientation of the erasing movement. In addition, we note the removal of surface residues, visible in particular in the top-left corner as the removal of dark-stained regions (white dashed line).

The qualitative assessment of the bone specimens through digital microscopy reveals several types of modifications to the bone surfaces after eraser use. First, the friction caused by the repetitive movement of the eraser during EEM results in the removal of residues and particles from the highest portion of the topography of the bone surfaces of 9 out of 12 ROIs (Fig. 3, Supplementary Fig. S2, Supplementary Table S3), which, in the case of 3 specimens, is also observable at the macroscopic scale. In contrast, the particles trapped within the lower portion of the topography do not seem to be removed during the limited duration of the experiment. We note that the removed residues will most likely get trapped within the eraser wastes generated during sampling. They therefore represent a potential source of contamination during the proteomic extraction of the organic matter present on the surface of a bone, in case these residues are proteinaceous in nature. Secondly the movement of dust, sediment particles and/or bone residues on the surface associated with the pressure applied during EEM sampling results in the formation of multiple linear features. Although we do not observe modification of the gross features of the bone surfaces after EEM through digital microscopy, we do observe the generation of parallel and regular micro-striations in the direction of the eraser movement on the control area of CC7-379 (Fig. 3). These modern striations are comparable to ancient use-wear traces^63,64^ but are unrelated to use of the bone. No other specimens exhibit micro-striations at the scale observed through digital microscopy.

### Quantitative microscopy

**Figure 4:**
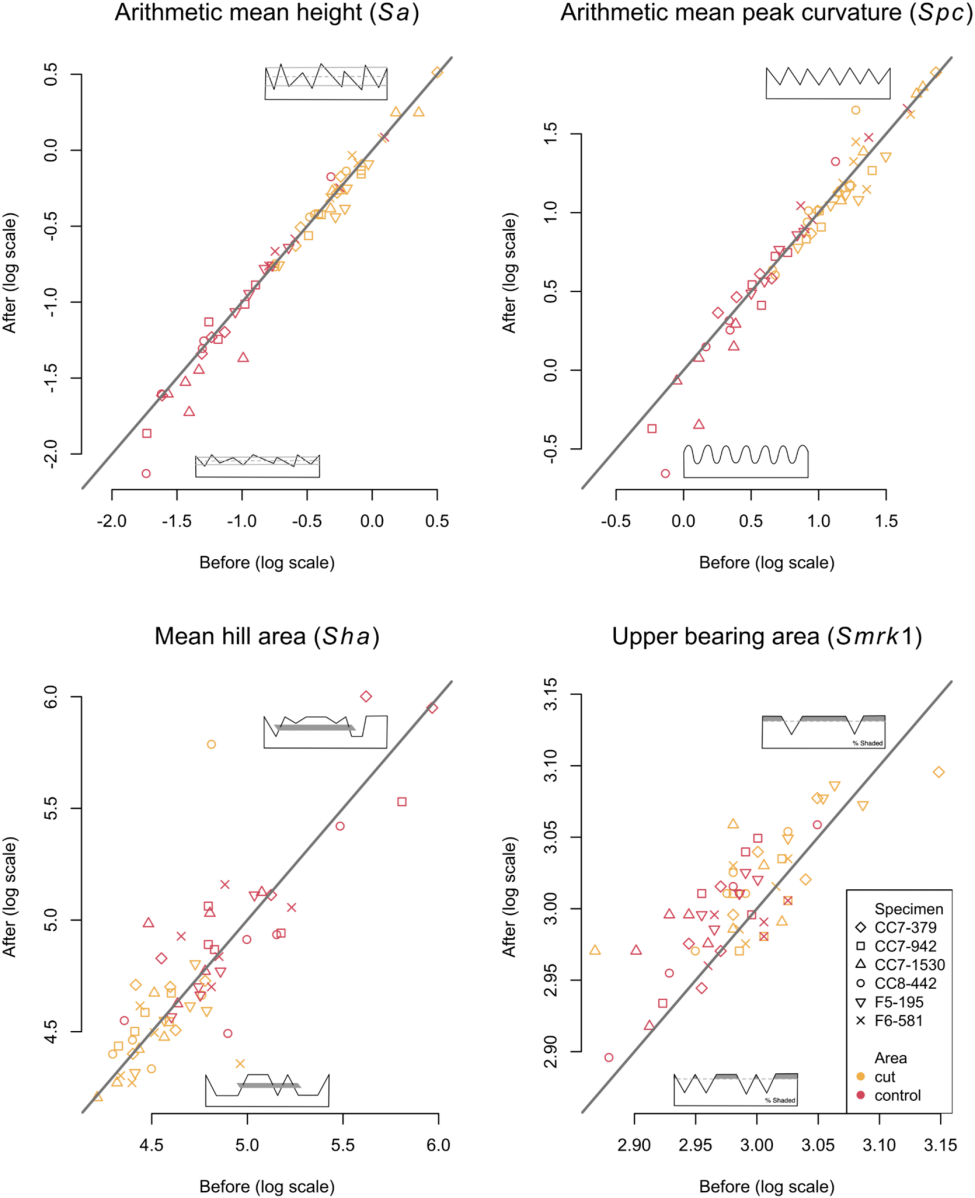
Matched before and after EEM pairwise scatterplot for each ISO 25178 surface texture parameter measured in this study (*Sa, Spc, Sha* and *Smrk1*). Lines represent equivalent parameter values after and before EEM. Each specimen is represented by different symbols, cut areas are in yellow and control areas are in red. 2D depictions of low and high values for each surface texture parameter are indicated on each plot.

Model predictions for the four ISO 25178 surface texture parameters indicate no distinguishable differences in the bone microtopography after EEM for three of the parameters (*Sa*, *Spc*, and *Sha*) (Table 1). Ratios of the estimated after-to-before parameter values that are close to 1 indicate that the before and after state are very similar (Fig. 4; Table 1). It should be noted that a small amount of variation in the parameters is expected due to the difficulty of repositioning the surfaces on the micrometer scale required. Even so, the estimated before and after EEM values for *Sa* and *Spc* are most often very similar and indicate that the surfaces retain their overall roughness and curvature of the surface peaks after use with the eraser. Though model predictions for *Sa* and *Spc* are overall statistically indistinguishable, there appears to be a modest trend for lower *Sa* and *Spc* values after EEM, which is associated with a greater number of eraser movements (Supplementary Fig. S8). We postulate that if sampling were to occur over a longer duration, this would result in a more substantial decrease in both *Sa* and *Spc*. While the overall predicted values for *Sha* also appear to be similar after EEM, the ratio of the estimated differences for this parameter has the largest credibility interval of the four tested ISO 25178 parameters (Table 1). Further, empirical observations of the matched before and after pairwise scatterplots indicate some degree of variation, including a number of outliers (Fig. 4). Because this variation scatters relatively equally around the 45-degree line and not to one side of it, these differences are either unrelated to eraser sampling or are the result of unpredictable and irregular surface alterations. If the latter, hill area as calculated by standard default settings used for the calculation of *Sha* may not be an appropriate settings for assessing microscopic bone alterations and need to be re-adjusted.

In contrast to *Sa*, *Spc* and *Sha*, model predictions for *Smrk1* indicate that there is an increase in values after EEM resulting in a larger portion of material in the peaks of the surfaces (Table 1). This shift in predicted values demonstrates that eraser use uniformly wears the highest areas, or hills, of the surface, causing a general flattening or plateauing of the uppermost part of the surface microtopography (Fig. 4). A comparison of the observed before and after differences with both the number of eraser movements and the amount of force exerted during EEM, shows a slight trend for greater before and after differences in association with both variables (Supplementary Figs. S8 and S9). However, we observe an absence of correlation between sample weights and the after to before differences of the four ISO 25178 parameters indicating that the mass of the samples collected (consisting of eraser wastes) had no influence on surface texture measurements (Supplementary Fig. S10). Though the increase in *Smrk1* is generally observed for most of the surfaces, the differences are more pronounced for those in the control area (Fig. 4). Because this parameter is a proportion of the surface material, the surfaces with less material [i.e., those with less surface roughness (< *Sa*)] are altered at a greater relative rate.

### Comparison of quantitative and qualitative microscopy

**Figure 5:**
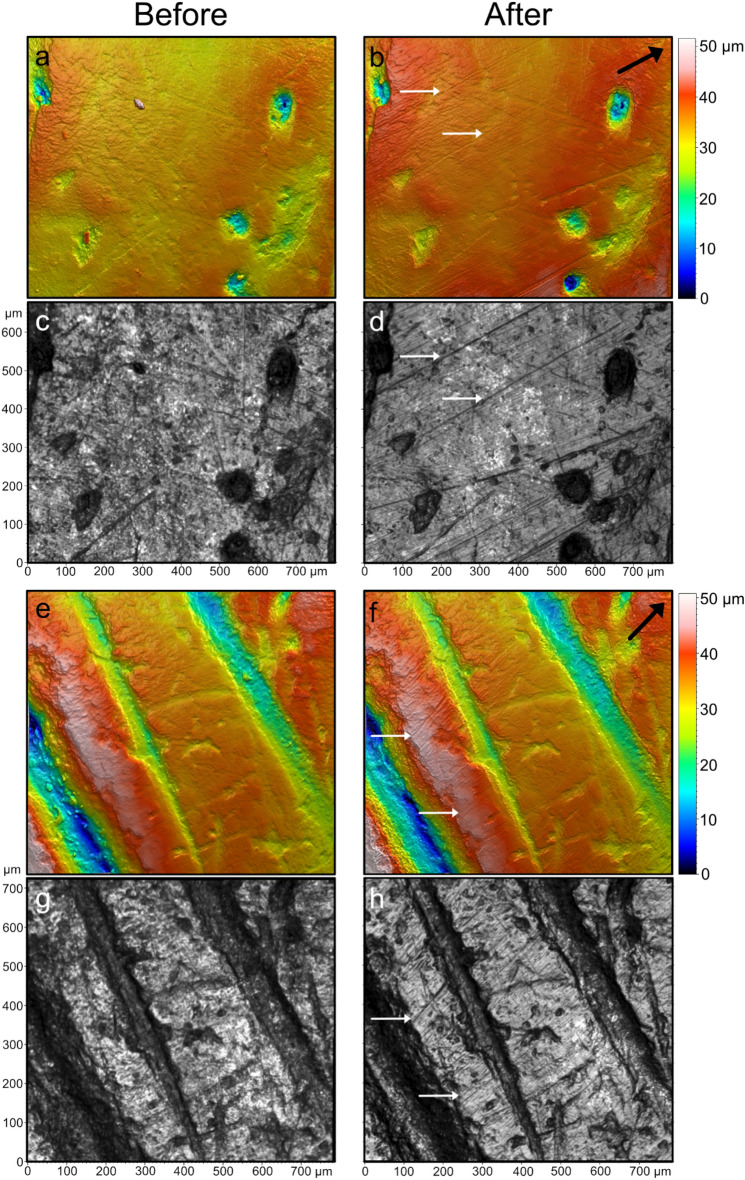
Bone surface microtopography of the specimen CC7-379, before (left) and after (right) EEM, using confocal disc-scanning microscopy. 3D surface models (**a**,**b**,**e**,**f**) and 2D intensity micrographs (**c**,**d**,**g**,**h**) of control (**a**–**d**) and cut areas (**e**–**h**). Orientation of eraser movements are indicated by the black arrows. Depth of the bone microtopography is color-coded with blue indicating the lowest valleys and white the highest peaks. We note the generation of microstriations after the use of EEM with some examples indicated by white arrows. We note the presence of a residue in the middle of the bone surface (**a**) which has been removed with the use of EEM (**b**) and is potentially related to the formation of the deeper traces located near its initial position.

A qualitative assessment of the bone surfaces through confocal microscopy supports the observations made using digital microscopy. At this higher magnification, we observe both “surface cleaning” and subparallel micro-striations oriented in the direction of eraser movement. Whereas digital microscopy reveals striations on one surface area of specimen CC7-379, visual inspection of the 2D intensity micrographs produced through confocal microscopy demonstrates that these surface modifications are present for every bone specimen, though not within each scanning location (Fig. 5; Supplementary Fig. S11). When visually comparing the same surfaces in both two and three dimensions (2D, 3D), these micro-striations or furrows appear to be superficial and do not alter the overall features of the bone surfaces (Fig. 5). The combined qualitative observations at different scales along with the quantitative increase of *Smrk1* indicates that EEM on bone creates friction that cleans the bone surface, while flattening the microtopography and creating fine micro-striations or furrows, which also causes the bone surface to appear polished at a macroscopic scale. The combination of multiple techniques and the assessment of surfaces at different scales is then crucial in order to obtain a comprehensive picture of the bone surface alterations after EEM.

## Discussion

Bone surface modifications provide the opportunity to associate an artefact retrieved from a Palaeolithic site with human occupation and activity, and potentially subsequent taphonomic processes at such archaeological sites. Therefore, the preservation of these modifications is crucial for the future analysis of such a specimen. With the development of non-destructive proteomic methods allowing for species determination, it is important to characterise the potential effects on bone surfaces. The Eraser Extraction Method constitutes one of the so-called non-destructive sampling techniques, and is based on the electrostatic extraction of proteinaceous molecules through repetitive movement of a soft Polyvinyl Chloride eraser directly on a bone surface^13,33,37^.

Our analysis of bone surfaces after EEM shows, overall, neither major modifications of the bone topography nor the removal of features, such as cut marks, at a macroscopic level. This is supported by the similar before and after values obtained for three of the four tested ISO 25178 parameters (*Sa*, *Spc*, and *Sha*) through quantitative microscopy. The relatively short duration of EEM appears to have little to no effect on these variables at the scale studied in this experiment. However, a similar analysis at higher magnification or with a larger selection of ISO parameters may have provided different results. Even so, the repetitive movement of the eraser on these bone surfaces generated several permanent modifications at the µm-scale that should be acknowledged prior to the use of this sampling technique on any archaeological bone.

A measurable increase in one of the ISO 25178 parameters (*Smrk1*) provides compelling evidence that EEM on bone flattens the surface. Given the relationship between the shift in *Smrk1* values and both the number of eraser movements and the force applied, it is likely that eraser sampling for a duration longer than 2 min, or with greater pressure, would further alter the bone surfaces. A previous study on experimentally worked bone surfaces found a similar pattern associated with duration of use when bones were worn against fresh animal skin, a supple, sticky material that readily incorporates external particles adding to its abrasiveness^48^. Another experimental study testing the effects of cleaning procedures on stone tools also found an increase in this surface texture parameter after rubbing dirt off of a flint flake for about 1 min^65^. As with fresh skin and sediment particles, this increase in *Smrk1* indicates that eraser use causes alterations to the highest portion of bone surfaces resulting in the plateauing of the microtopography at the µm-scale. This pattern is most likely the result of friction generated during EEM combined with microscopic particles such as dust or calcite crystals wearing the surface when pressed against the bone and dragged by eraser movement. Further, this mechanical action likely led to the formation of the micro-striations or furrows observed on the bone specimens at multiple scales, which has also been observed on lithic artifacts^66^. Therefore, the effect of the EEM on bone surface microtopography could be related to the abrasiveness and size of the particles present on the bone surfaces during sampling.

We interpret the increase in *Smrk1* after EEM as an explanation for the qualitative observation that eraser use appears to clean and remove residues preserved on bone surfaces. This observation in both 2D and 3D microscopy represents an irreversible pattern. This implies that any potential traces of substances, such as adhesives, pigments, organic residues or residue traces, can potentially be removed from the surface, which in turn could prevent any subsequent residue analysis seeking to address the function of the worked piece^67^.

Although the presence of multiple micro-striations are only observed on a single specimen (CC7-379) through visual inspection using digital microscopy, they are measurable on each studied specimen at higher magnification using confocal disc-scanning microscopy. When looking at potential variables that could have influenced the creation of these traces and their appearance at different magnifications, we note the similarity in the force exerted and the number of eraser movements applied to the cut and control areas of the specimen CC7-379. At lower magnification using digital microscopy, the control area exhibits micro-striations after eraser use, while the cut area does not. This discrepancy cannot be explained by differences in force applied or eraser movements. However, it should be considered that the standardised parameters used in this experiment may have not permitted the complete visualisation of these surface alterations through digital microscopy, and variation in bone inclination along with differing oblique light orientations might have provided a clearer shadow effect and better assessment of the bone surfaces at that scale^68^. Nonetheless, the standardised protocol presented in this study allowed for the identification of various surface alterations caused by the use of EEM on palaeolithic bone specimens.

These observed micro-striations are comparable to surface modifications produced either during the use of a bone as a tool or during other taphonomic processes, and are observed at different magnifications using distinct microscopic methods^46,48,63,64^. While the observations made on the 2D intensity micrographs show clear and well-defined striations, they appear more superficial within the 3D surface texture. If such a bone was subsequently studied without a detailed sampling record, a functional analysis could lead to misinterpretation of such traces^69^. Worse, this sampling method applied to a bone tool could overprint any ancient use-wear traces indicative of the tool’s function, obscuring interpretation. Thus, if one chooses to use EEM on bone, it would be important to incorporate this method into a phased approach, one in which EEM should be conducted subsequent to any functional analyses. The application of EEM on other mineralized and non-mineralized tissue surfaces might generate similar modifications, which is something that should be investigated prior to future applications of this technique. Indeed, our results emphasize the importance of maintaining a detailed record associated with this extraction technique, similar to any protocol for destructive sampling. This is especially important for future analyses that have not been anticipated.

Moreover, the exclusive capture of low-molecular weight peptides and the low signal-to-noise ratio of the three dominant peptide markers limits the opportunity to obtain a discriminant species assignment for taxonomic groups not separable based on low-mass peptide markers. As a result, it can be expected that EEM, and other eZooMS approaches, result in a potentially low success rate when applied to Palaeolithic bone specimens^13,37^.

Thus, the bone surface alterations and the potential low success rate of the eZooMS analysis using EEM highlighted in this paper should bring caution to the use of this extraction method on Palaeolithic faunal assemblages, and especially worked bones such as bone tools. The creation of modern alterations to the surfaces of archaeological specimens unrelated to their fabrication or use should be avoided to prevent subsequent misinterpretations.

## Conclusion

The taxonomic assessment of fragmented or heavily modified bone artefact specimens represents an ongoing problem, especially in Palaeolithic archaeology. The recent development of non-destructive extraction techniques has opened up the possibility to contribute to the understanding of hominin behaviour related to the manufacture and use of such objects. To understand the impact of such biomolecular sampling methods on Palaeolithic bones, we performed a controlled sampling experiment measuring applied force in addition to qualitative and 3D quantitative microscopy prior to and after the use of the EEM. Overall, while the EEM can be used on Palaeolithic bone objects, it provides low-quality MALDI-TOF MS spectra and modifies bone surfaces. These modifications include aspects mimicking use-wear traces, and involve striations on all bone surfaces. Although the gross features of the bone microtopography remain, the quantitative differences shown by one of the four tested ISO 25178 surface texture parameters (*Smrk1*) indicates a general flattening of the bone surface. Based on our results, we conclude that the EEM should not be considered as a non-destructive sampling method when applied on Palaeolithic bone surfaces. Further work is therefore required to overcome sampling limitations for the analysis of worked bones. We recommend that such development should be done in a manner that takes into account the analysis of bone surfaces via other methods, including functional analyses aimed at interpreting use-wear traces and residues.

## Supplementary Information

Supplementary Information.

Supplementary Table S1.

Supplementary Table S3.

## Data Availability

All original, unfiltered surface texture scans, surface texture templates (MountainsMap) and raw data for bayesian modeling (R and stan) used in this study are stored at the Edmond database of the Max Planck Society (MPS, Munich, Germany); and can be accessed via Edmond: 10.17617/3.6z.
