# Supplementary material for: The effect of eraser sampling for proteomic analysis on Palaeolithic bone surface microtopography
Source: Sci Rep. 2021 Dec 8;11:23611. doi: 10.1038/s41598-021-02823-w (PMC8655045; doi:10.1038/s41598-021-02823-w)
Supplement: Supplementary file 1 — Supplementary Information. [file 41598_2021_2823_MOESM1_ESM.docx]

##### **Supplementary Information to:**

##### **The effect of eraser sampling for proteomic analysis on Palaeolithic bone surface morphology**

Virginie Sinet-Mathiot^1*^, Naomi L. Martisius^1,2^, Ellen Schulz-Kornas^1,3^, Adam van Casteren^1^, Tsenka Tsanova^1^, Nikolay Sirakov^4^, Rosen Spasov^5^, Frido Welker^6^, Geoff M. Smith^1^, Jean-Jacques Hublin^1,7^

1 - Department of Human Evolution, Max Planck Institute for Evolutionary Anthropology, Leipzig, Germany.

2 - Department of Anthropology, The University of Tulsa, Tulsa, OK, USA.

3 - Department for Cariology, Endodontology and Periodontology, University of Leipzig Medical Center, Leipzig, Germany.

4 - National Institute of Archaeology with Museum, Bulgarian Academy of Sciences, Sofia, Bulgaria.

5 - Archaeology Department, New Bulgarian University, Sofia, Bulgaria.

6 - Section for Evolutionary Genomics, Globe Institute, University of Copenhagen, Copenhagen, Denmark.

7 - Collège de France, Paris, France.

*Corresponding author: Virginie Sinet-Mathiot (virginie_sinet@eva.mpg.de)


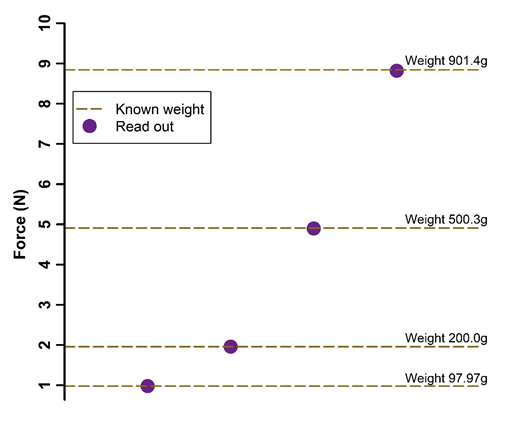


Supplementary Figure S1: The load cell was calibrated using a series of known weights. A weight was placed on the instrumented stage and the readout recorded for 15 seconds. If the readout matched (within the 0.05% precision range) what was expected for the known weight in Newtons, then the load cell was considered calibrated correctly. If they differed, then recalibration was required, and the calibration factor adjusted until the readout aligned with what was expected for the known weight.


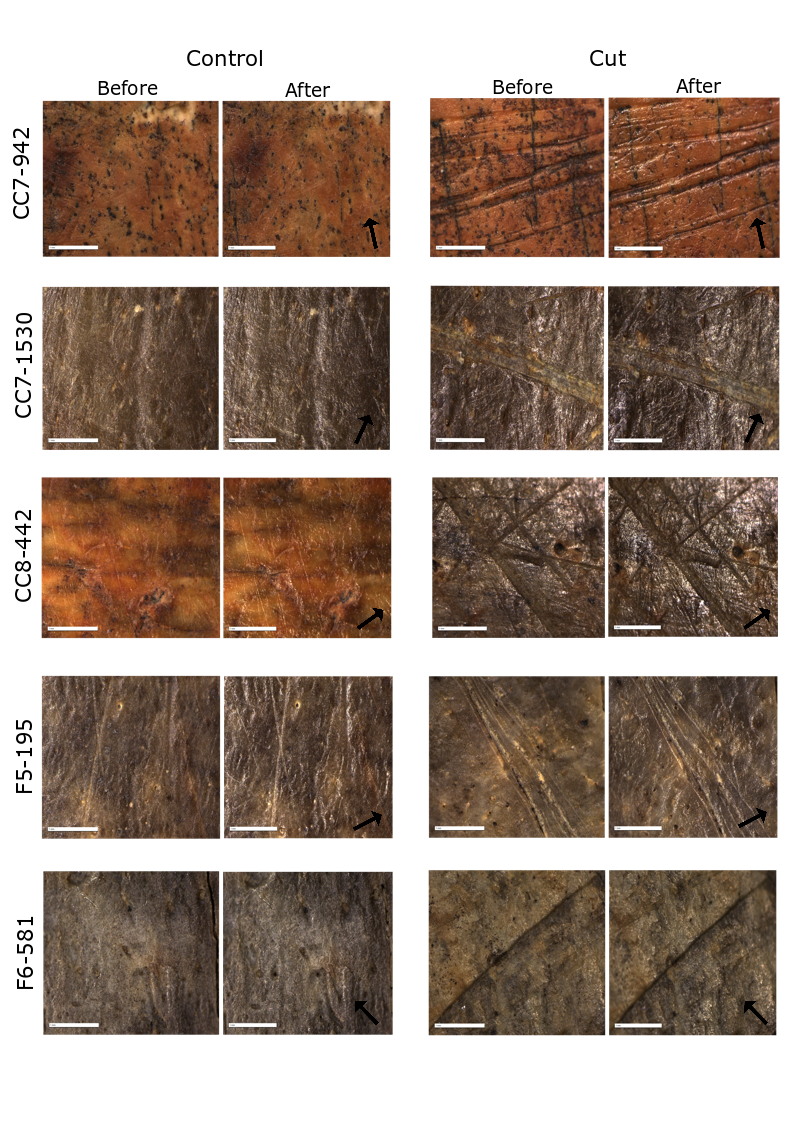


Supplementary Figure S2: Micrographs of the control and cut areas of all studied specimens using automated digital microscopy (ZEISS, Smartzoom 5, magnification x106), before (left) and after (right) EEM. The scale represents 1cm on each image. Black arrows indicate the orientation of the eraser movements during the experiment. We note an increase of the bone surface shininess in the case of 9 ROIs out of 12, generally located on the highest areas or hills of the surface topography after the use of EEM, resulting from the repetitive movement of the soft eraser rubbing the bone surface. This reflectivity mimics the visual pattern of polished surfaces which one would expect to observe on a bone that has been used or handled repetitively.


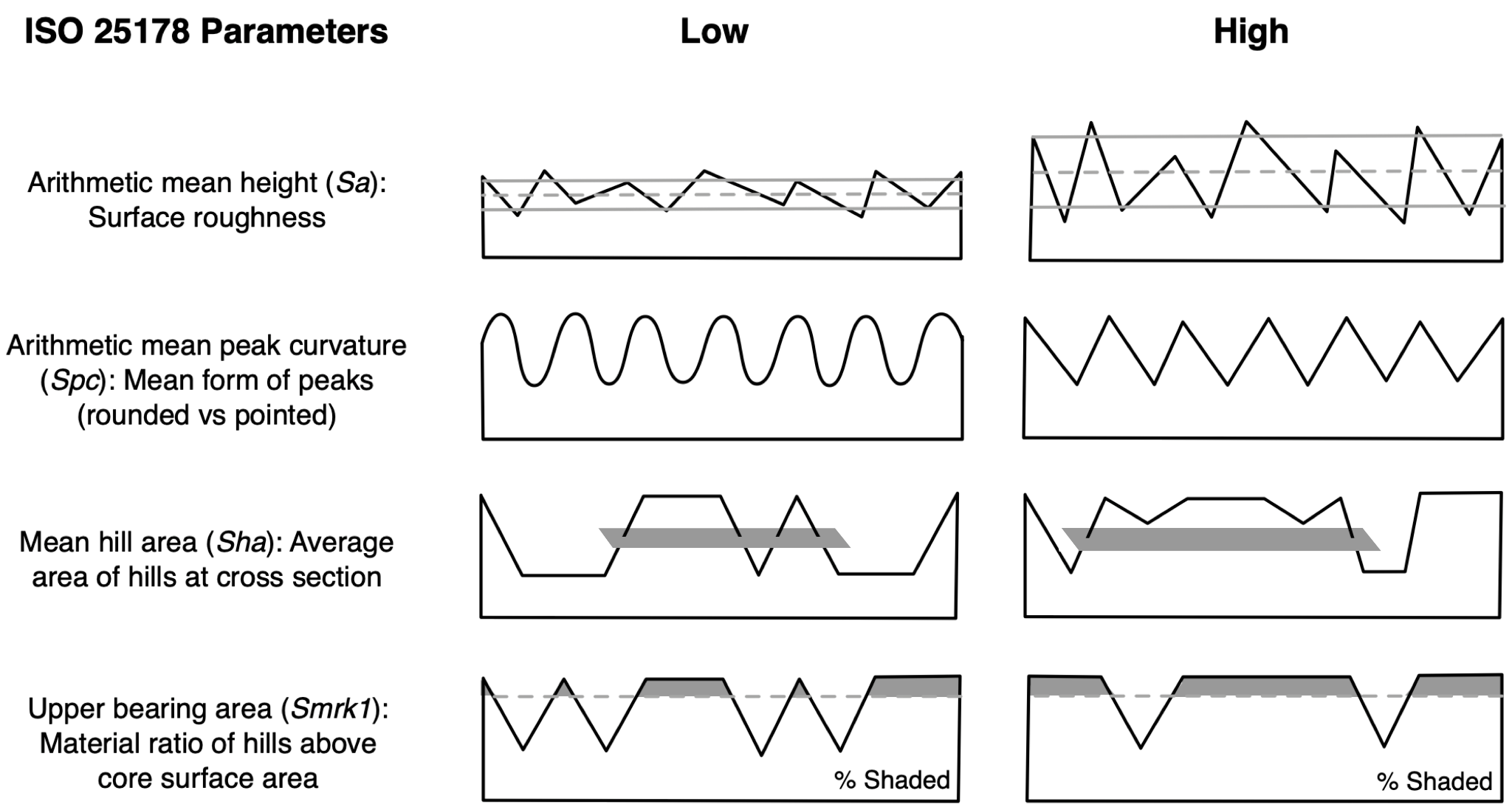


Supplementary Figure S3: 2D depictions of surface texture parameters (ISO 25178) (*Sa, Spc, Sha and Smrk1*) indicating low and high values (adapted/modified from ^1–3^).

| Model | Effects | Design matrix | Δ elpd | Δ se |
| --- | --- | --- | --- | --- |
| M1 | 2 fixed + random | Area + erasing + specimen + measurement location + error | 0 | 0 |
| M0 | 1 fixed + random | Area + specimen + measurement location + error | -15.5 | 7.2 |

Supplementary Table S2: Model effects including their representation in each model’s design space and LOO expected log predictive density (elpd) and standard error (se) differences relative to M1.


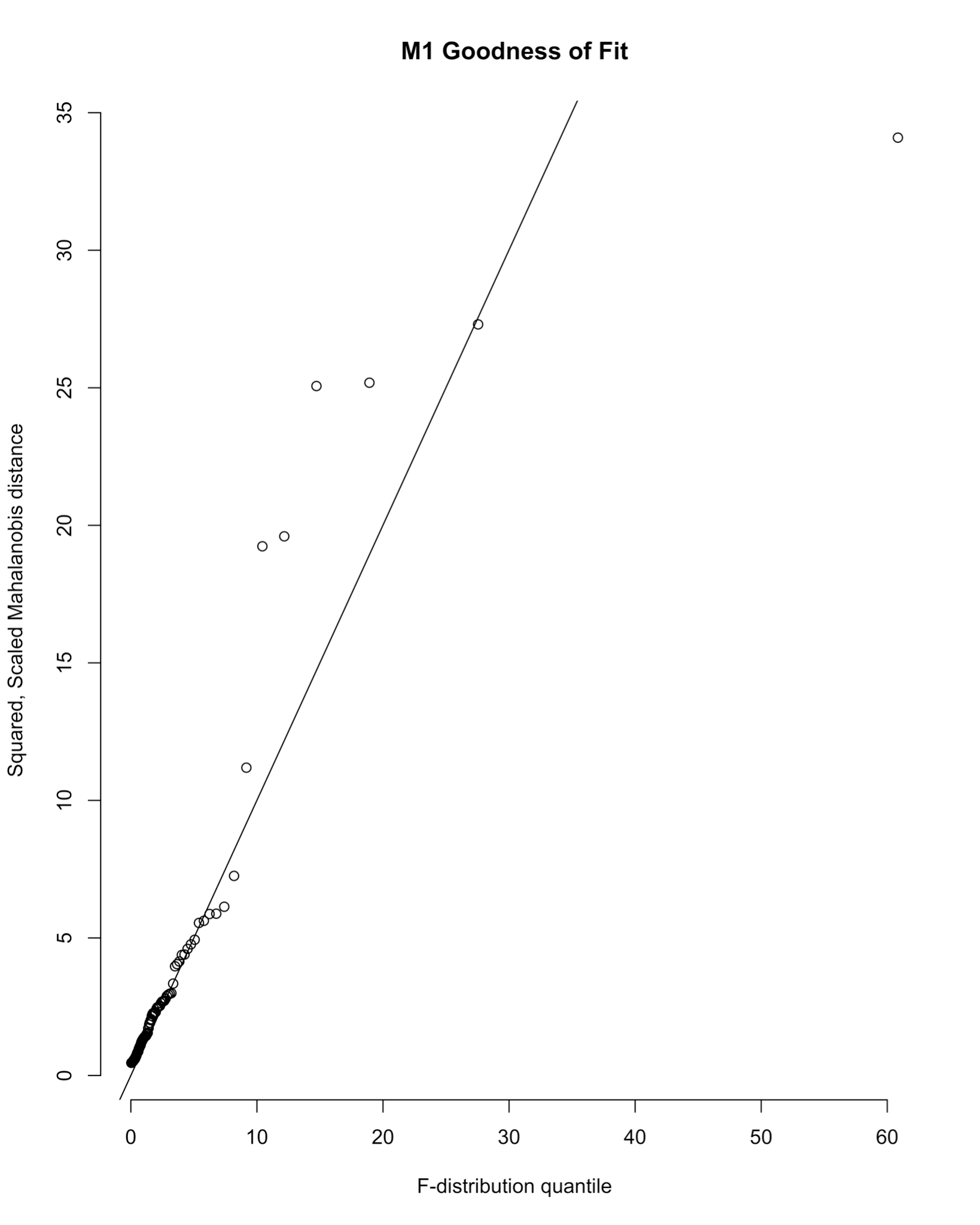


Supplementary Figure S4: Quantile-quantile plot of scaled and squared Mahalanobis distances (between observations and their predicted values) versus theoretical quantiles of the F- distribution ^4^. Most observations follow the theoretical quantiles well.

#####
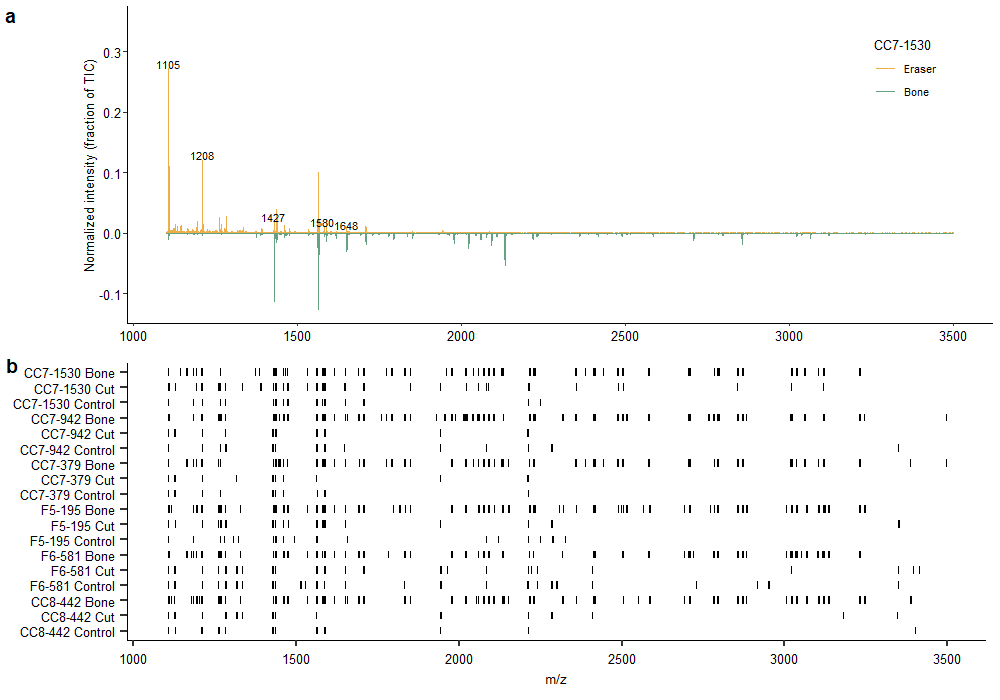


Supplementary Figure S5: a) Example of MALDI-TOF mass spectra obtained from the eraser extraction of a bone surface (in orange) and from the buffer extraction of a bone sample (in green) of CC7-1530. The intensity is normalised as a fraction of the TIC for better comparison. b) Gel view representation of the spectra obtained for each specimen from the buffer extraction of a bone sample (Bone) and the eraser extraction of the Control and Cut area. Each bar represents a peak with a S/N ratio of 5 or higher. This representation highlights the systematic absence of high-molecular weight peptide markers within the spectra obtained from the eraser extraction of bone surfaces.


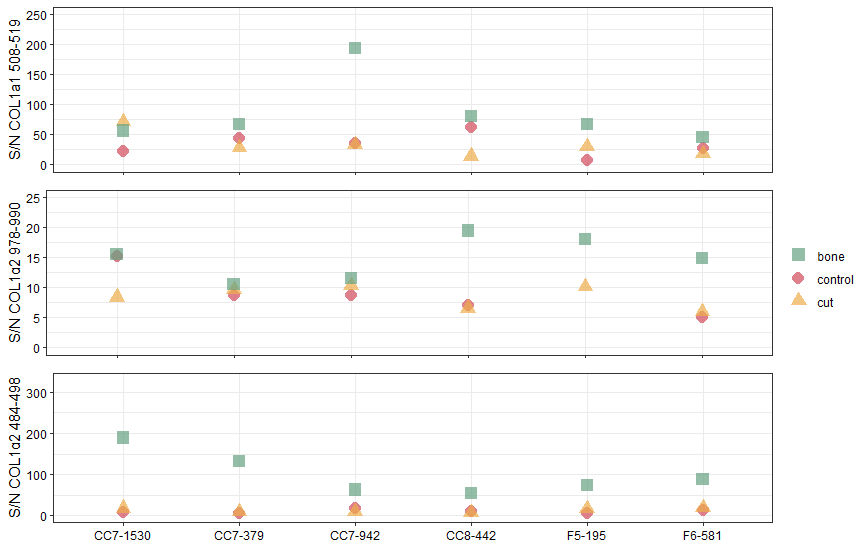


Supplementary Figure S6: signal-to-noise (S/N) ratio for peptide markers COL1α1 508-519, COL1ɑ2 978-990, and COL1ɑ2 484-498 for each specimen. In green are the values obtained for each bone sample, in yellow are the values for each cut area sampled with an eraser and in red are the values measured for each control area sampled with an eraser.


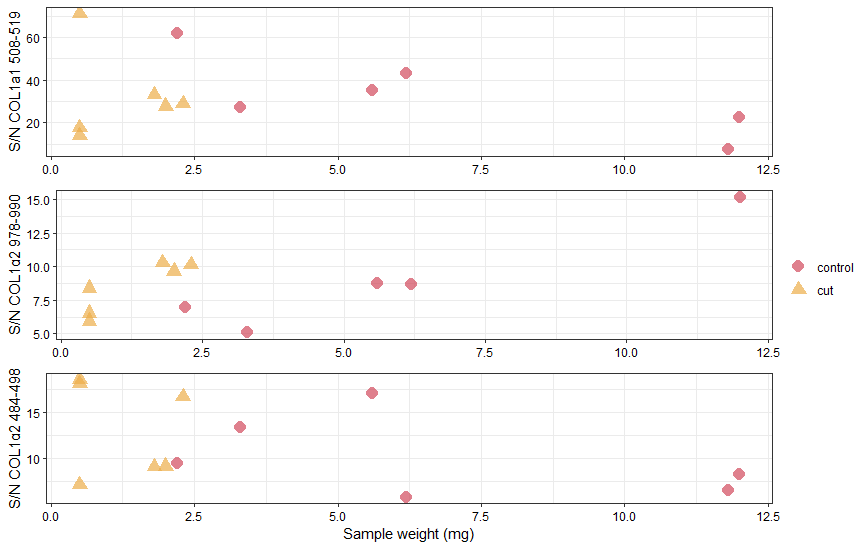


Supplementary Figure S7: Signal-to-noise (S/N) ratio for peptide markers COL1α1 508-519, COL1ɑ2 978-990, and COL1ɑ2 484-498 across sample weight generated during each EEM event. In yellow are the values obtained for each cut area sampled with an eraser and in red are the values measured for each control area sampled with an eraser.

#####


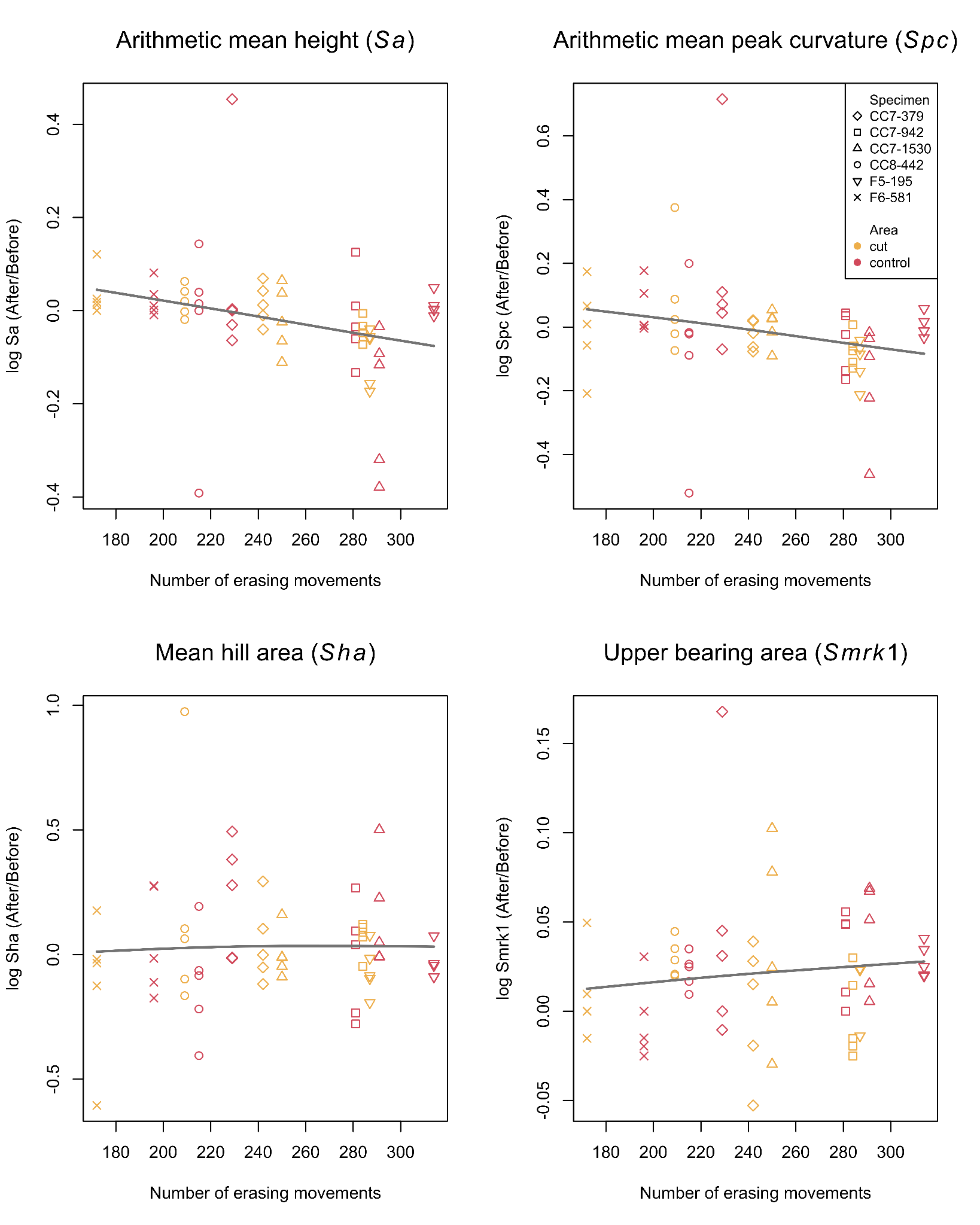


Supplementary Figure S8: After to before differences of the four ISO 25178 parameters (*Sa, Spc, Sha* and *Smrk1*) by the number of eraser movements. Each specimen is represented by different symbols, cut areas are in yellow and control areas are in red.


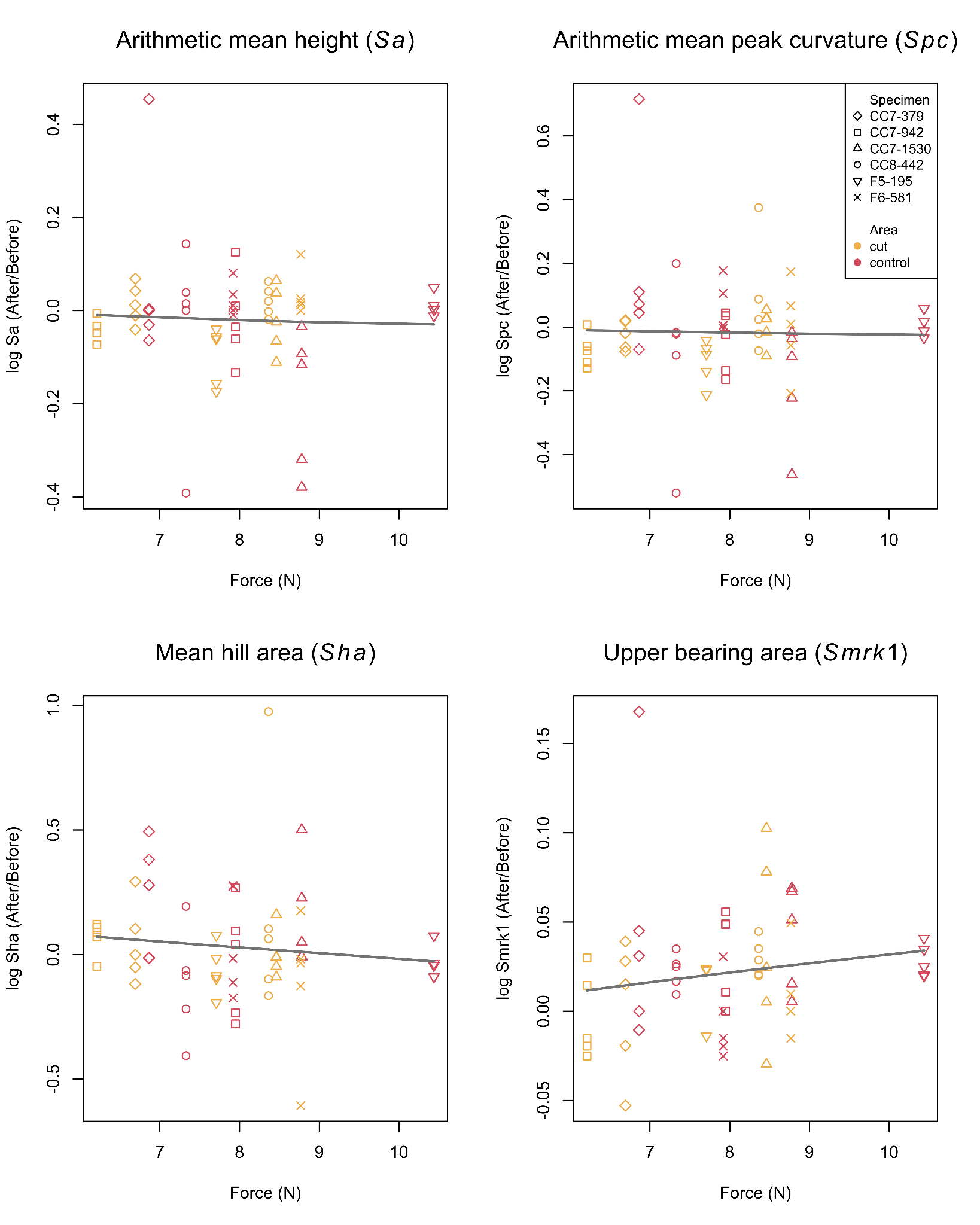


Supplementary Figure S9: After to before differences of the four ISO 25178 parameters (*Sa, Spc, Sha* and *Smrk1*) by force (in Newton). Each specimen is represented by different symbols, cut areas are in yellow and control areas are in red.


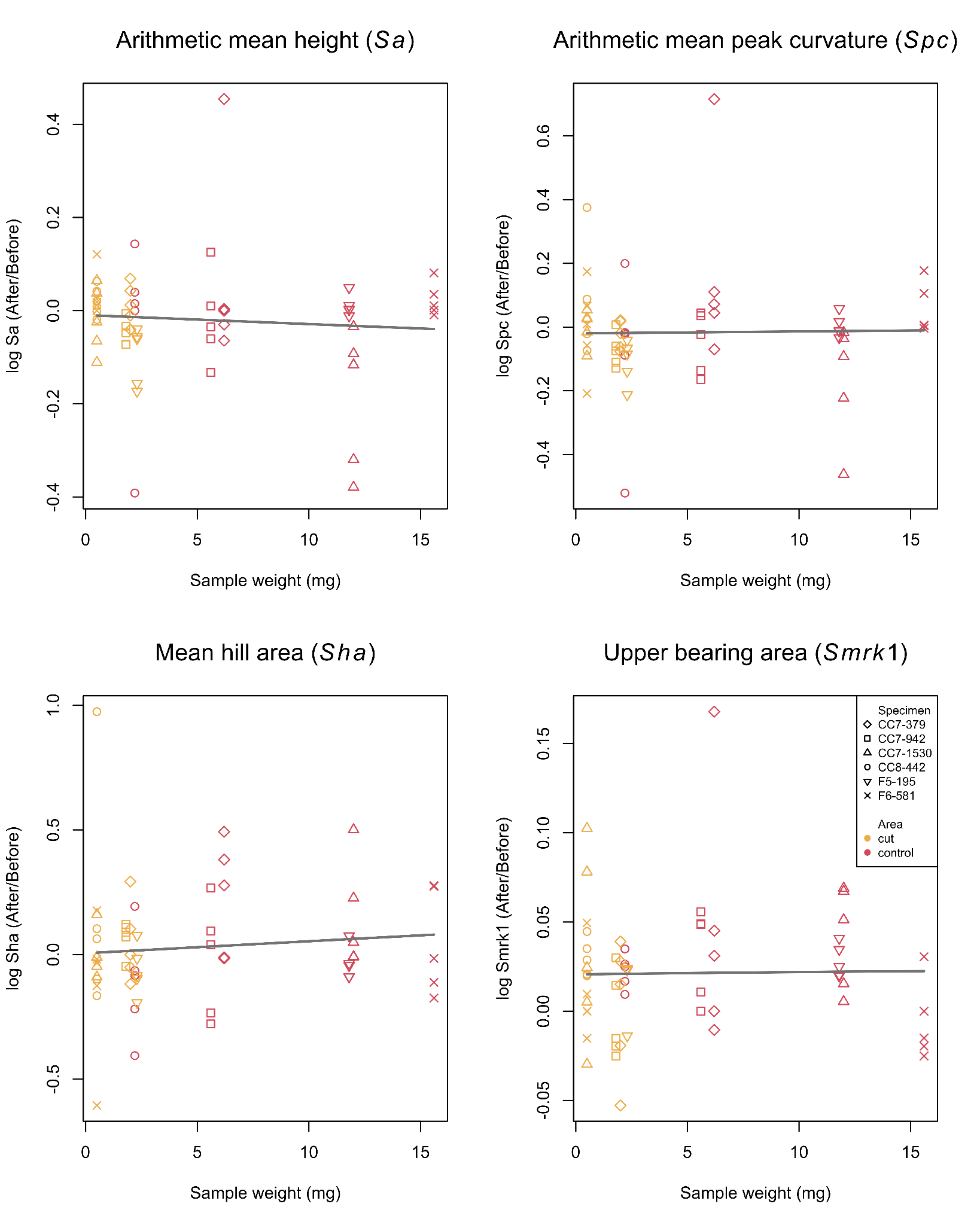


Supplementary Figure S10: After to before differences of the four ISO 25178 parameters (Sa, Spc, Sha and Smrk1) by sample weight (in mg). Each specimen is represented by different symbols, cut areas are in yellow and control areas are in red.


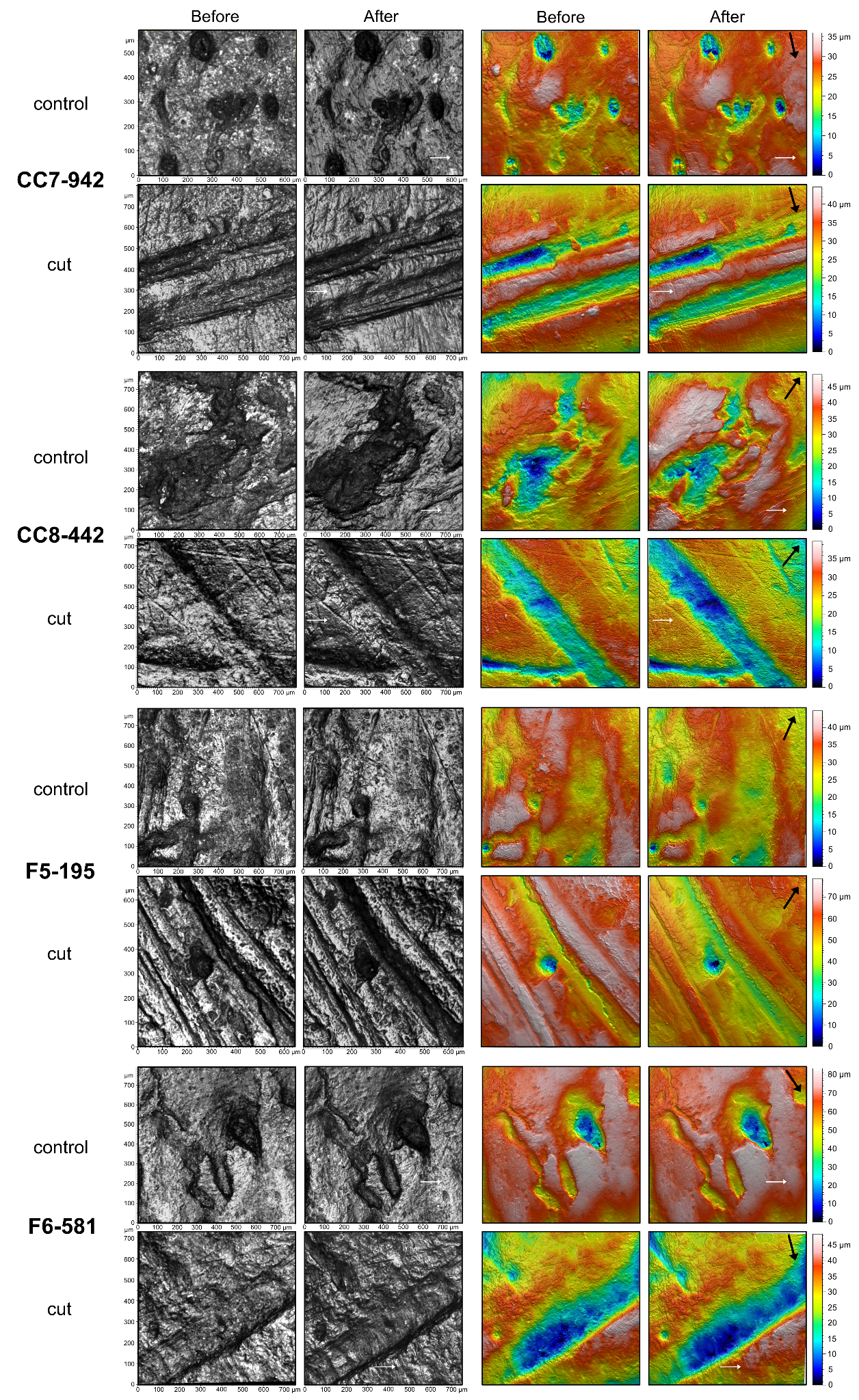


Supplementary Figure S11 : 2D intensity images (left) and 3D models (right) of the bone surface microtopography of four specimens (CC7-942, CC8-442, F5-195, F6-581), before (left) and after (right) EEM in both control (upper) and cut (lower) areas. Orientation of the eraser movements are indicated by the black arrows. Depth of the bone microtopography is color-coded with blue indicating the lowest valleys and white the highest peaks. We note the generation of microstriations after the use of EEM with some examples indicated by the white arrows.

Supplementary Table S1: Raw ISO 25178 data before and after EEM for each surface texture parameter on each ROIs of all specimens. Sa is expressed in µm, Spc in 1/µm, Sha in µm2 and Smrk1 in %

**.xlsx file attached**

Supplementary Table S3: Sample information for all bone specimens included in this study and analysed through destructive sampling and EEM (control and cut area), including sample weights, average peak forces, number of erasing movements, taxonomic identifications obtained through ZooMS, peptide marker masses (rounded to whole m/z values), signal to noise ratio (S/N) for the three dominant peptide markers and qualitative observations through digital microscopy.

**.xlsx file attached**

References:

1. Kaiser, T. M., Clauss, M. & Schulz-Kornas, E. A set of hypotheses on tribology of mammalian herbivore teeth. *Surf. Topogr.: Metrol. Prop.* **4**, 014003 (2016).

2. Martisius, N. L. *et al.* Time wears on: Assessing how bone wears using 3D surface texture analysis. *PLoS One* **13**, e0206078 (2018).

3. Martisius, N. L., McPherron, S. P., Schulz-Kornas, E., Soressi, M. & Steele, T. E. A method for the taphonomic assessment of bone tools using 3D surface texture analysis of bone microtopography. *Archaeol. Anthropol. Sci.* **12**, 251 (2020).

4. Roth, M. *Linköping university electronic press report: On the multivariate t distribution*. (2013).
